# Chymase Mediates Injury and Mitochondrial Damage in Cardiomyocytes during Acute Ischemia/Reperfusion in the Dog

**DOI:** 10.1371/journal.pone.0094732

**Published:** 2014-04-14

**Authors:** Junying Zheng, Chih-Chang Wei, Naoki Hase, Ke Shi, Cheryl R. Killingsworth, Silvio H. Litovsky, Pamela C. Powell, Tsunefumi Kobayashi, Carlos M. Ferrario, Andras Rab, Inmaculada Aban, James F. Collawn, Louis J. Dell'Italia

**Affiliations:** 1 Birmingham Veteran Affairs Medical Center and Division of Cardiovascular Disease, Department of Medicine, University of Alabama at Birmingham, Birmingham, Alabama, United States of America; 2 Teijin Institute for Bio-Medical Research, Teijin Pharma Ltd, Tokyo, Japan; 3 Department of Pathology, University of Alabama at Birmingham, Birmingham, Alabama, United States of America; 4 Wake Forest University, Winston Salem, North Carolina, United States of America; 5 Department of Cell, Developmental, and Integrative Biology, University of Alabama at Birmingham, Birmingham, Alabama, United States of America; 6 Department of Biostatistics University of Alabama at Birmingham, Birmingham, Alabama, United States of America; University of Pecs Medical School, Hungary

## Abstract

Cardiac ischemia and reperfusion (I/R) injury occurs because the acute increase in oxidative/inflammatory stress during reperfusion culminates in the death of cardiomyocytes. Currently, there is no drug utilized clinically that attenuates I/R injury in patients. Previous studies have demonstrated degranulation of mast cell contents into the interstitium after I/R. Using a dog model of I/R, we tested the role of chymase, a mast cell protease, in cardiomyocyte injury using a specific oral chymase inhibitor (CI). 15 adult mongrel dogs had left anterior descending artery occlusion for 60 min and reperfusion for 100 minutes. 9 dogs received vehicle and 6 were pretreated with a specific CI. In vivo cardiac microdialysis demonstrated a 3-fold increase in interstitial fluid chymase activity in I/R region that was significantly decreased by CI. CI pretreatment significantly attenuated loss of laminin, focal adhesion complex disruption, and release of troponin I into the circulation. Microarray analysis identified an I/R induced 17-fold increase in nuclear receptor subfamily 4A1 (NR4A1) and significantly decreased by CI. NR4A1 normally resides in the nucleus but can induce cell death on migration to the cytoplasm. I/R caused significant increase in NR4A1 protein expression and cytoplasmic translocation, and mitochondrial degradation, which were decreased by CI. Immunohistochemistry also revealed a high concentration of chymase within cardiomyocytes after I/R. In vitro, chymase added to culture HL-1 cardiomyocytes entered the cytoplasm and nucleus in a dynamin-dependent fashion, and promoted cytoplasmic translocation of NR4A1 protein. shRNA knockdown of NR4A1 on pre-treatment of HL-1 cells with CI significantly decreased chymase-induced cell death and mitochondrial damage. These results suggest that the beneficial effects of an orally active CI during I/R are mediated in the cardiac interstitium as well as within the cardiomyocyte due to a heretofore-unrecognized chymase entry into cardiomyocytes.

## Introduction

Following an acute myocardial infarction, reperfusion of the myocardium is a required event after the ischemic insult for salvaging viable myocardium. Unfortunately, reperfusion causes further cardiomyocyte injury and death [1]. Limiting reperfusion injury is an important objective to improve myocardial salvage and currently there is no effective therapy for this in the clinical setting.

Resident mast cell (MC) degranulation is an early event in ischemia/reperfusion (I/R) injury. MCs normally exist in the myocardium in an intact form but become activated in response to acute stress, rapidly releasing enzymes and hormonal mediators into the cardiac interstitium [2]. Chymase is a family of chymotrypsin-like serine proteases stored in secretory granules of MCs, and in the heart, there is a disproportionately larger amount of chymase protein compared to its mRNA. In mast cell-deficient mice, there is no detectable chymase mRNA, suggesting that at baseline in the absence of any stress the mast cell is the primary source of chymase [3]. In addition to converting angiotensin I (Ang I) to Ang II [3], [4], chymase directly degrades fibronectin [5], activates matrix metalloproteinases (MMPs) [6], [7], [8], and initiates apoptosis in cardiomyocytes through disruption of the focal adhesion complex *in vitro* [9], [10]. Based on amino acid sequence homology, chymase is divided into two groups, α and β. Only α-chymase has been identified in human and dog, while α and multiple isoforms of β-chymase are present in rodents [11]. In the current study, we utilize a dog model to test whether using a specific oral chymase inhibitor [12] in the interstitial fluid (ISF) space attenuates cardiomyocyte injury during I/R.

In a pig model of I/R, treatment with an orally active chymase inhibitor reduces infarct size, suggesting that chymase may play a central role in I/R injury [13]. To better understand the consequences of I/R injury on heart damage, we performed a gene array on LV tissue undergoing I/R injury and the mRNAs from the I/R region and the non-I/R regions were directly compared. The most dramatically affected gene within the I/R region was the nuclear receptor subfamily 4A1 (NR4A1). NR4A1 is a transcription factor whose translocation from nucleus to cytoplasm results in mitochondria-induced apoptosis in a variety of cells types in response to extracellular stresses [14]–[16]. Here we show that chymase inhibition blocks NR4A1 upregulation and translocation to the cytoplasm. Furthermore, through a combination of *in vivo* and *in vitro* studies, we demonstrate that chymase enters the cardiomyocyte cytoplasm and nucleus during I/R, implying that chymase may have both extracellular and intracellular targets in the dog heart that mediate I/R injury.

## Methods

### Ethics Statement

This study was carried out in strict accordance with the recommendations in the Guide for the Care and Use of Laboratory Animals of the National Institutes of Health (NIH publication No. 85–23 m, revised 1996). The protocol was approved by the Institutional Animal Care and Use Committee (IACUC) at the University of Alabama at Birmingham (APN: 130808903).

### Antibodies

For the dog immunohistochemistry: TUNEL staining was performed using the DeadEnd Fluorometric TUNEL System (Promega, WI) according to the manufacturer's instructions. Antibodies used included laminin (chicken polyclonal 1∶100, Abcam #ab-14055, MA), cardiac myosin, heavy chain (mouse monoclonal 1∶100, Abcam #ab-15, MA), NR4A1/Nur77 (rabbit polyclonal 1∶100, Santa Cruz Biotechnology #sc-5569, TX), desmin (mouse monoclonal 1∶200, Abcam, MA), and chymase (rabbit polyclonal 1∶400, Bioss Antibodies #bs2353R, MA).

For the HL-1 immunocytochemistry: Antibodies used included cardiac myosin, heavy chain (mouse monoclonal 1∶100, Abcam #ab-15, MA), NR4A1/Nur77 (rabbit polyclonal 1∶100, Santa Cruz Biotechnology #sc-990, TX), chymase (mouse monoclonal 1∶50, Abcam #ab-2377, MA), and caveolin 3 (rabbit polyclonal 1∶250, Abcam #ab-2912, MA).

### Experimental Protocol

Adult mongrel dogs with a weight range of 25–30 kg were used in this study. Dogs were maintained under a deep plane of isoflurane anesthesia and were mechanically ventilated (Harvard Apparatus, MA). After an intravenous bolus of heparin (300 IU/kg), a fluoroscopically-guided balloon (3×20 mm) was inflated (6 atmospheres) immediately distal to the first diagonal branch of the left anterior descending coronary artery. After 60 min of left anterior descending artery occlusion, the balloon was deflated with reperfusion for 100 min. I/R was induced in 15 adult mongrel dogs. Six were pretreated with a specific α-chymase inhibitor [17] (TEI-F00806, 100 mg/kg, bid) for 5 days before I/R induction. Nine were treated with placebo (vehicle controls). Five dogs without treatment and I/R induction, and these served as normal controls.

### Dog Sacrifice

After 60-min ischemia and 100-min reperfusion, the hearts were quickly arrested with saturated KCl, excised, and tissues were subsequently collected.

### Cell Lines

HL-1 cells were cultured and maintained as described with some modifications [18]. Briefly, the cells were grown in the flasks coated with gelatin/fibronectin, and maintained in Claycomb Medium (SAFC Biosciences, Sigma) supplemented with 10% fetal bovine serum, 0.1 mM norepinephrine, 100 U/ml Penicillin and 100 ug/ml Streptomycin and 2 mM L-glutamine (Sigma-Aldrich, MO) [18]. The medium was changed every 24 h, and the cells were grown at 37°C in 5% CO_2_ and 95% air.

### Analysis of Interstitial Fluid (ISF) for Angiotensin-Converting Enzyme (ACE) and Chymase Activities

**Figure 1:**
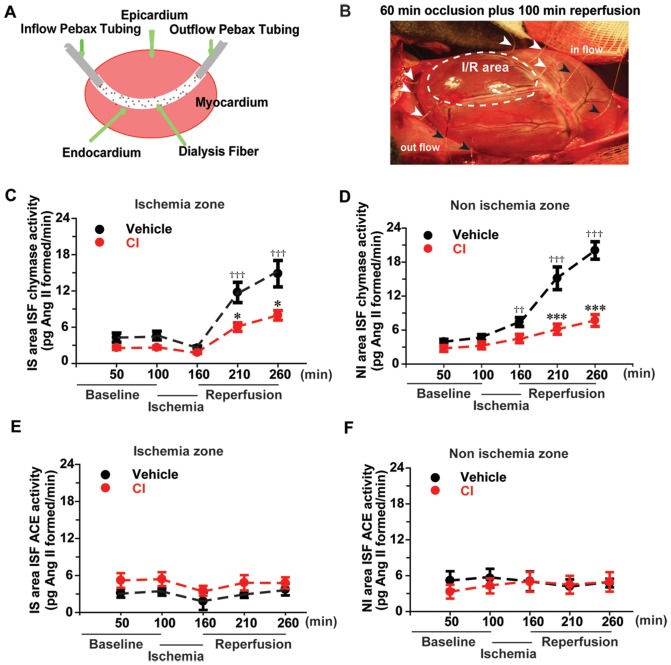
*In vivo* cardiac microdialysis demonstrates increased ISF chymase activity during I/R that is decreased by pretreatment with chymase inhibitor (CI). Panel a demonstrates the concept of cardiac microdialysis and panel b demonstrates the three microdialysis probes located in the area of I/R region (white arrows) and the two probes in the non-I/R region (black arrows) in the open chest dog heart. Panels c and d demonstrate ISF chymase activity in I/R zone (c) and non-I/R zone (d) in the Veh (black) and CI (red) pretreated dogs. Panels e and f demonstrate ISF ACE activity in the I/R (e) and non-I/R zones (f) in Veh and CI dogs. The vehicle chymase activity increased during reperfusion and therefore the analysis compared the vehicle baseline with increasing chymase activity during reperfusion. ^††^ *p*<0.01 vehicle baseline vs. vehicle during ischemia;^†††^ *p*<0.0001 vehicle baseline vs. vehicle during reperfusion; * *p*<0.05, *** *p*<0.001 vehicle vs. CI treated dogs. Veh dog, n = 9. CI dog, n = 6.

Microdialysis probes were positioned in the LV (Figure 1a and b): three probes in I/R and two in non I/R zones (arrowheads) for ISF infusion of chymase-selective substrate [Pro^11^, DAla^12^] Ang I (2.5 µM) and angiotensin-converting enzyme (ACE)-selective substrate [Pro^10^] Ang I (0.5 µM) as previously described [3]. The probe effluents were collected for chymase and ACE activity analysis at 5 time points: 50 min and 100 minutes after probe insertion and before the left anterior descending artery (LAD) ligation, during the 60 min of the LAD ligation (160 min time point), and 50 and 100 min during reperfusion (210 and 260 time points, Figure 1c–f).

### Analysis of Gene Expression by Microarray and Quantitative real-time RT-PCR

Total RNA was extracted using Qiagen RNeasy Fibrous Tissue Mini Kit (Qiagen Sciences, MD) and treated with Ambio TURBO DNA-free DNase. Samples with OD ratio 260/280 >1.8, 28S/18S >1.5 were sent to Beckman Coulter Genomics (NC) for microarray processing with Agilent one color 4×44K canine cDNA array. Comparative analysis of expression profiles was carried out using Genespring GX 11.0 (Agilent Technologies, CA). Microarray data were validated by RT-PCR as previously described [19].

### Western Blot Analysis

Western blot analysis was performed on the tissue lysates. LV mid tissue was homogenized in RIPA buffer containing EDTA, protease and phosphatase cocktail inhibitors (all from Thermo Fisher Scientific, IL). Lysates (10–40 µg) were separated on a 4–20% gradient Bis/Tris gel (Invitrogen, CA) by sodium dodecyl sulfate (SDS)-polyacrylamide gel electrophoresis, transferred to a PVDF membrane and probed with primary antibodies overnight (4°C) then with appropriate horse-radish-peroxidase-conjugated secondary antibodies (1∶2000–1∶5000). Bands were visualized by enhanced chemiluminescence (Thermo Fisher Scientific, IL), scanned and quantitated in Scion Image v4.0.3.2 (Scion Corporation), and normalized to tubulin.

### Chymase and Transferrin Uptake in HL-1 Cells

For the endocytosis experiments with chymase (Sigma-Aldrich, MO) and transferrin-Alexa Fluor 594 (Life Technologies, OR), the cells were grown for one day in 4-chamber culture slides (BD Falcon, BD Biosciences, MA) coated with gelatin/fibronectin. The medium was removed and replaced with serum-free medium in 1.0% DMSO (vehicle) or 160 µM dynasore (Selleckchem, MA) for a 30 min preincubation period. Chymase (2.5 µg/ml) or transferrin-Alexa 594 (Life Technologies, OR) was added to the medium for an additional 60 min at 37°C. The cells were then chilled on ice, washed three times with PBS (for the transferrin-alexa 594 uptake experiments) or PBS with 0.5 M NaCl added (for the chymase uptake experiments), and then processed for immunofluorescence as described below.

### Immunofluorescence Analyses

Immunohistochemistry was performed on formalin-fixed paraffin-embedded LV tissue from normal, vehicle- and CI-treated dogs. 5 µm tissue sections were mounted on positive charge (+) slides, deparaffinized in xylene, and rehydrated in a graded series of ethanol. To visualize apoptosis in cardiomyocytes, the DeadEnd Fluorometric TUNEL System Kit (Promega, WI) was used, followed with laminin primary antibody (1∶100, Abcam, MA) and Alexa Fluor 594-conjugated secondary antibody (1∶500, Invitrogen, OR) staining for myocyte identification. After heat-induced antigen retrieval was performed with citrate buffer (Vector Laboratories, CA), other tissue sections were blocked with 5% normal serum (in 1% bovine serum/PBS), followed by overnight incubation at 4°C with primary antibodies as described above.

Immunocytochemistry was performed on HL-1 cells treated with chymase or transferrin-Alexa Fluor 594 as described above. The chymase treated cells were fixed in 3% formaldehyde (Tousimis, Rockville, MD) for 20 min at room temperature (RT) and washed 3 times in PBS, and permeabilized with 0.1% Triton-X-100 (Fisher #BP-151) for 20 min at RT. 10% normal serum (in 1% bovine serum/PBS) for 1 hr at RT was used for blocking, followed by overnight incubation at 4°C with primary antibody as described above. For both sets of experiments, Alexa Fluor 488- and 594-conjugated secondary antibodies (1∶500, Life Technologies/Invitrogen, OR) with the appropriate host combinations were used to stain for visualization, as indicated in figure legends. Nuclei were stained (blue) with DAPI (1.5 µg/ml; Vector Laboratories, CA). Image acquisition was performed on a Leica DM6000 epifluorescence microscope (Leica Microsystems, IL) with SimplePCI software (Compix, Inc., PA). Images were adjusted appropriately to remove background fluorescence.

### Serum Cardiac Troponin Analysis

Serum troponin I (cTnI) concentration was determined by ELISA kit from Life Diagnostics Inc., PA.

### Cardiomyocyte Isolation and Chymase Treatment

Cardiomyocytes were isolated from the tissue by recirculating perfusion buffer supplemented with 2 mg/mL collagenase type II (Invitrogen, CA) as previously described in our laboratory [17]. Isolated cardiomyocytes were plated on laminin-coated plates. Cells without treatment served as the controls.

### Knockdown of NR4A1 in Chymase-treated HL-1 Mouse Cardiomyocytes

HL-1 cells were transfected with p-GFP-ShNR4A1 and scrambled p-GFP-ShRNA (OriGene, MD) for 5 days and then treated with 5 µg/ml chymase for 2 h. Mitochondrial apoptosis was detected by Abcam Apoptosis Detection Kit (Abcam, MA). In healthy cells, the dye (MitoCapture) accumulates and aggregates in the mitochondria, giving off a red fluorescence. In apoptotic cells, the dye does not aggregate in the mitochondria since these mitochondria do not maintain an active membrane potential. In this case, the MitoCapture dye remains in the cytoplasm and fluoresces a weak green. Apoptotic cells were counted on whole chamber area at ×20 objective and normalized by number of normal cells.

### Transmission Electron Microscopy (TEM) Analysis

TEM was performed by EMLABS, Inc, Birmingham, AL. Heart tissue was fixed overnight in 2.5% glutaraldehyde in 0.1 M sodium cacodylate buffer (Electron Microscopy Sciences, PA). After post-fixation with 1% osmium tetroxide in 0.1 M cacodylate buffer, tissue was dehydrated with a graded series of ethanol and embedded in Epon resin. Semi-thin (0.5 µm) and ultra-thin (90 nm) sections were cut, mounted on copper grids, and post-stained with uranyl acetate and lead citrate. Sections were viewed at 60 kv with a Philips 201 transmission electron microscope (FEI Co., OR) for qualitative changes in ultrastructure.

### Statistical Analysis

Data are presented as mean ± standard error of mean (SEM). In the in vivo dog studies of ISF chymase and ACE activities, we compared (1) treatment groups at each time point of 50, 100, 160, 210 and 260 minutes, and (2) times 100, 160, 210, and 260 minutes relative to the 50 minute baseline for each region (IR and non-IR) and each outcome (ISF chymase and ACF activities). We performed separate analyses for each outcome and each region. In each analysis, we defined a group consisting of 10 categories containing two treatment groups and 5 time points. To address the correlation between repeated measures within a dog, a general linear model was utilized for repeated measures with unstructured covariance for analyzing ACE activity and a mixed model for chymase activity. Instead of using the traditional cut off of p<0.05, a stricter cut off of p<0.025 was used to determine significant pairwise comparisons to minimize inflating type I error rates. For the microarray, a list of genes with ≥2-fold change was generated first. The list was then filtered by “Benjamin and Hochberg false discovery test” as previously described in our laboratory [19]. Genes with a false discovery of less than 0.05 were used to form the final list of differentially expressed genes for further analyses. Validation of NR4A1 and ATF3 from the microarray was performed using a Mann-Whitney unpaired t test. All other analyses were performed using an unpaired t test. A p value of <0.05 was used to determine significance.

## Results

In the analysis of the ISF from the I/R treated dogs, microdialysis probes were positioned in the LV (Figure 1a and b): three probes in the I/R and two in non-I/R regions (arrowheads). The probes were then used for ISF infusion of chymase- and ACE-Ang I specific substrates as described previously [3]. Initial examination of the ISF chymase activity indicated that it remained relatively unchanged during 60-min of ischemia (Figure 1c and d). However, chymase activity increased 3- to 4-fold after 100-min of reperfusion in both I/R and non-I/R LV regions, which indicating that there was chymase activity in non-I/R LV ISF. Pretreatment with the CI significantly decreased this effect (Figure 1c and d). During the same time course, the ISF ACE activity remained unchanged (Figure 1e and f) in both vehicle- and CI-treated dogs. The heart rate, systolic and diastolic blood pressures in vehicle- and CI-treated dogs were not affected during the 5 days of CI pretreatment, but the systolic and diastolic pressures did decrease with CI at the end of I/R protocol compared to the vehicle-treated dogs (Table S1).

**Figure 2:**
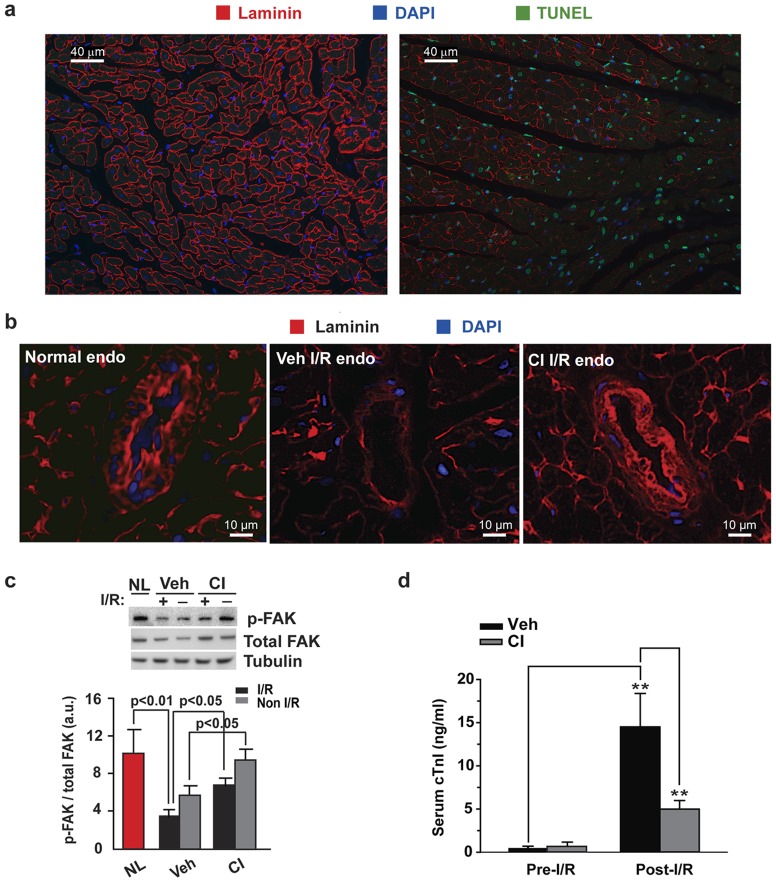
CI decreases laminin degradation and attenuates I/R injury in the dog heart. (a) Compared to the normal LV (left panel), I/R results in a decrease in the laminin staining in this border region (right panel). There is a greater increase in apoptotic cells (green) with loss of laminin. (b) CI treatment decreased I/R induced laminin degradation. (c) FAK dephosphorylation is markedly decreased in normal (NL) dog LV compared to I/R LV. CI treatment attenuated I/R induced p-FAK dephosphorylation. (d) CI pretreatment significantly attenuates the I/R-induced release of cTnI into the circulation. ** *p*<0.01. NL dog, n = 3; Veh dog, n = 6; CI dog, n = 5.

To determine the damage caused by chymase after I/R, we examined 3 known indicators of cardiac damage after I/R: laminin, focal adhesion kinase (FAK), and troponin I. Laminins are a part of the basement membrane that surrounds cardiomyocytes and blood vessels, and are important for cell adhesion and survival [20]. FAK is recruited to participate in focal adhesion dynamics and the activated form is phosphorylated [21], [22]. Troponin I release in the serum is indicative of cardiomyocyte damage from injured and apoptotic cells in humans [23] and laboratory animals [24]. After I/R, there is progressive decrease in laminin staining in the I/R to that is adjacent a non-I/R LV endocardial region (Figure 2a, right panel). The demarcation between the I/R and non I/R region is clearly seen and should be compared to the normal control (NL, Figure 2a, left panel). TUNEL staining (shown in green) indicated that a large percentage of the cells in both regions were undergoing apoptosis (Figure 2a, right panel). In a larger view of an LV region with laminin surrounding a blood vessel, the laminin staining in the normal control (Figure 2b, left panel) is very bright. In the I/R region, however, the staining is largely lost (middle panel), whereas in the CI pretreated dogs the laminin staining is spared (right panel), indicating that chymase is somehow involved in this process. As another indicator, in both I/R and non-I/R regions of the LV, the phosphorylated levels of FAK (p-FAK) are dramatically decreased, and CI blunts the loss of p-FAK (Figure 2c). And in the final marker, the troponin I (cTnI) levels in the serum are barely detectable before I/R, whereas they are dramatically elevated afterwards and significantly decreased with CI (Figure 2d). All of these measures indicate that blocking chymase activity attenuates many of the damaging features of I/R injury.

**Figure 3:**
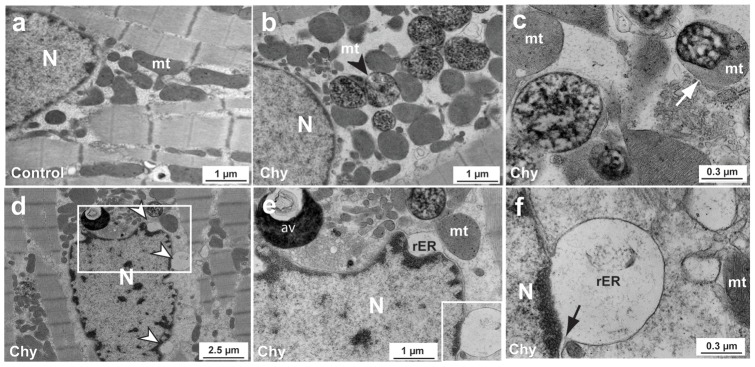
Chymase promotes cell detachment and death *in vitro* in laminin-plated dog cardiomyocytes. Control dog cardiomyocyte TEM is shown in the upper left panel (a) and images of chymase-treated cardiomyocytes (Chy) are shown in the other panels (b–f). Black arrowhead points to degraded mitochondria (mt, b). White arrow points to a partially degraded mitochondria (c). In bottom d–f panels, chymase is shown to induce the vacuolation of ER in the dog cardiomyocytes (white arrowheads, d). A blowup of the boxed area of the left panel (d) is shown in middle panel (e) and a larger blowup is shown in the right panel (f) further demonstrating the vacuolation of ER and autophagic vacuoles of degraded perinuclear mitochondria. Black arrow points to the membrane of ER and nucleus (f). mt, mitochondria; N, nucleus; rER, rough endoplasmic reticulum; av, autophagic vacuole.

To directly assess the effects of chymase on cardiomyocytes, adult dog cardiomyocytes were isolated and cultured on laminin-coated plates. Transmission electron microscopy (TEM) images of isolated dog cardiomyocytes in Figure 3 show degenerated mitochondria in the perinuclear area (panel b and c, arrow head and arrows), and vacuolation of the endoplasmic reticulum (panel d, white arrowheads) after 2 h of 5 µg/ml of chymase treatment. An expanded view of the endoplasmic reticulum stress is shown in panel e, which is expanded further in panel f with the markedly expanded ER merged to the outer nuclear membrane (black arrow). This TEM analysis indicates that the cardiomyocytes are undergoing rapid morphological changes and mitochondrial damage even after only two hours of chymase before evidence of cell detachment and death.

**Figure 4:**
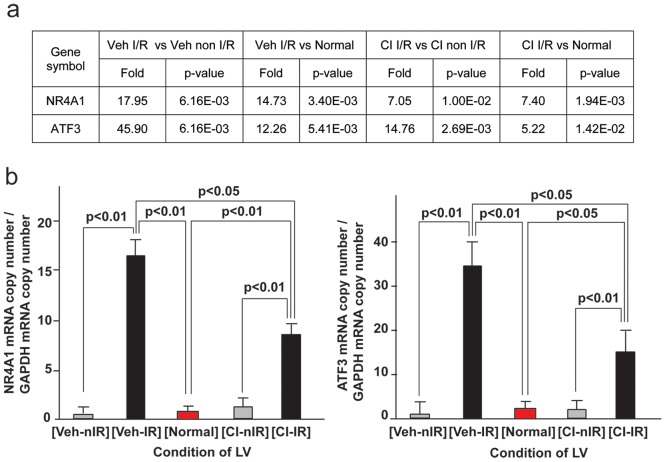
Regulation of NR4A1 mRNA in normal, vehicle and CI treated dog LV I/R and non-I/R regions by microarray and RT-PCR. Table (a) demonstrates Genesping GX.11 fold change from microarray and p value for mRNA intensity value of NR4A1 and ATF3 in normal, I/R and non-I/R vehicle- and CI-treated dog LV. Panel b demonstrates real-time RT-PCR validation of NR4A1 (left) and ATF3 (right) mRNA in normal, I/R and non-I/R vehicle- (Veh-nIR) and CI-treated dog LV. Normal dog, n = 5; Veh dog, n = 6; CI dog, n = 6.

In order to assess the effects of I/R in dog LV gene expression, a microarray analysis was performed on normal LV tissue and LV tissue from I/R and non-I/R LV regions that were vehicle treated or CI treated (Figure 4). The microarray analysis indicated that two transcription factors were dramatically upregulated during I/R, NR4A1 and activating transcription factor 3 (ATF3). The table shown in Figure 4a shows the microarray fold changes and p-values of these two genes. The mRNA levels of the two genes, NR4A1 and ATF3, were validated by real-time RT-PCR (Figure 4b) and the results indicated that NR4A1 and ATF3 were elevated more than 15- and 30-fold, respectively, in the vehicle treated I/R LV compared to the vehicle treated non-I/R and normal LV (Figure 4b). Pretreatment with CI significantly decreased NR4A1 and ATF3 mRNA in the I/R region.

**Figure 5:**
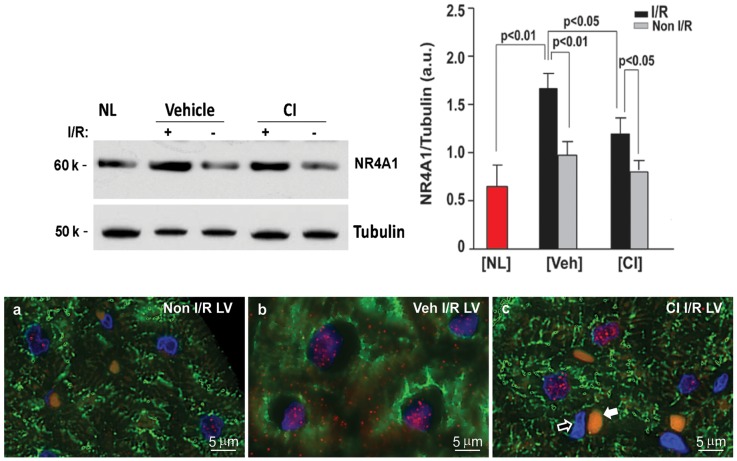
NR4A1 protein expression in normal, vehicle- and CI-treated LV. Upper panels show western blot and quantitation of NR4A1 in the I/R LV. The increase of NR4A1 protein in the I/R region is significantly attenuated by CI pretreatment. Lower panel a demonstrates very little NR4A1 (red) in the non-I/R LV region, while panel b demonstrates increased nuclear NR4A1 and its cytoplasmic migration in vehicle-treated I/R heart, but not in the CI-treated heart (panel c). Panel c also demonstrates the negative staining for NR4A1 in the endothelial cell nucleus (open arrow) that is located beside a red blood cell (white arrow). The yellowish color of red blood cell is caused by auto-fluorescence. Normal dog, n = 3; Veh dog, n = 6; CI dog, n = 5.

Given that NR4A1 and ATF3 are stress response genes, and that NR4A1 can induce mitochondrial-mediated apoptosis, we next tested CI could block this effect. To address this, two analyses were performed. In the first, western blot analysis was performed on the I/R and non-I/R LV tissue to monitor for NR4A1 expression. Compared to the normal LV tissue (NL Figure 5, upper panels), there was an increase in NR4A1 protein expression in the LV I/R region, which was significantly decreased by CI treatment. Analysis by immunohistochemistry confirmed that there was an increase in NR4A1 in the LV I/R but not in the non-I/R LV region (Figure 5b and c compared to 5a). Figure 5b also demonstrates that I/R induces translocation of NR4A1 from cardiomyocyte nuclei to the cytoplasm but not in the CI treated LV (Figure 5c). Figure 5c also demonstrates that NR4A1 is only expressed in cardiomyocytes and not in interstitial cells and this is exemplified by the negative staining for NR4A1 in endothelial cell nuclei (open arrow), which is located beside a red blood cell (white arrow).

**Figure 6:**
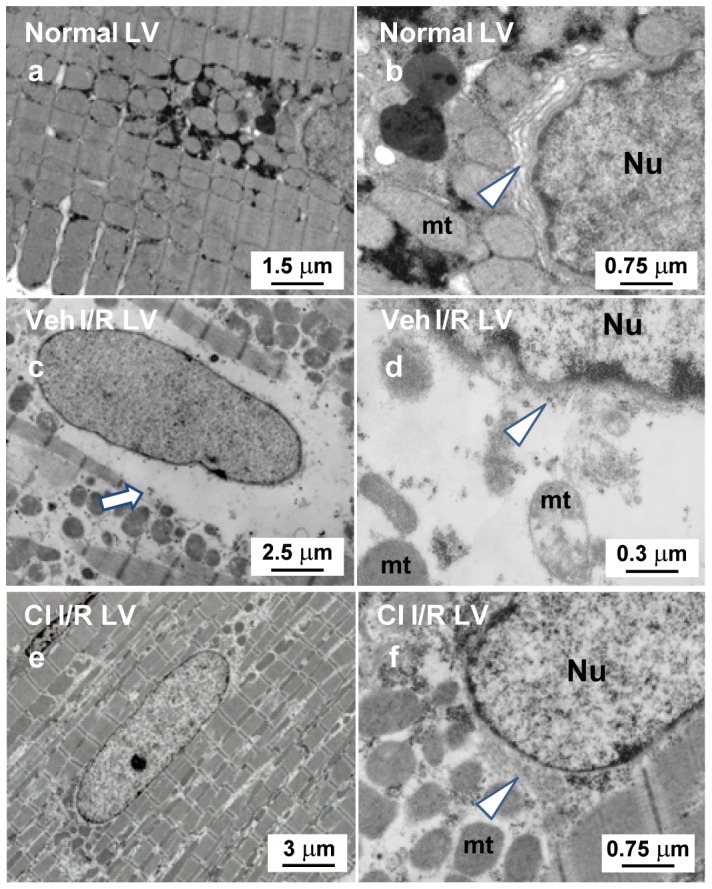
TEM demonstrate perinuclear degradation of endoplasmic reticulum (ER) and mitochondria degradation with I/R. TEM images demonstrate tightly packed distribution of perinuclear mitochondria (a) and at higher magnification the location of ER (b, white arrow head). Middle panels (c and d) demonstrate the perinuclear vacuole that is also present by immunohistochemistry in Figure 3d–f, demonstrating breakdown of perinuclear mitochondria and ER (white arrow head). Lower panels (e and f) demonstrate that degradation of ER and mitochondria are prevented by CI pretreatment. Nu: nucleus; mt: mitochondria

To study the connection between NR4A1 cellular distribution and cardiomyocyte endoplasmic reticulum (ER) and mitochondria disruption, TEM was performed on the LV tissue from normal, vehicle I/R and CI treated I/R LV tissue. Analysis by TEM indicates that the mitochondria and ER surrounding the nucleus are intact in the normal LV (Figure 6a and b). In I/R tissue, however, there are severe morphological changes to the mitochondria and ER resulting in abnormally large perinuclear empty spaces (Figure 6c and d). Pretreatment with CI dramatically attenuates these morphological changes in cardiomyocyte ER and mitochondria (Figure 6e and f).

**Figure 7:**
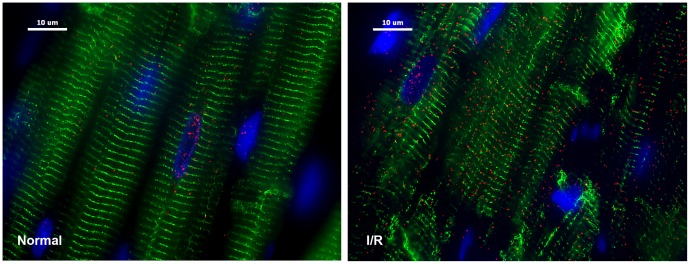
Chymase inside dog cardiomyocytes during ischemia/reperfusion (I/R). Adult dogs with 60 min of LAD occlusion and 100 min of reperfusion (right panel) and normal control (left panel). I/R LV demonstrates marked increase in chymase (red) with areas of breakdown of desmin (green, right) vs. normal control tissue (left). Blue: DAPI.

To determine the amount of chymase present in normal LV dog heart before I/R injury, the tissue was examined by immunohistochemistry. The results indicate that there is small amount of chymase in the interstitium and in the cardiomyocyte in normal heart tissue (Figure 7, left panel). However, there was a dramatic increase in the cytoplasmic and nuclear staining for chymase (Figure 7, right panel) after 60 min of ischemia and 100 min of reperfusion.

**Figure 8:**
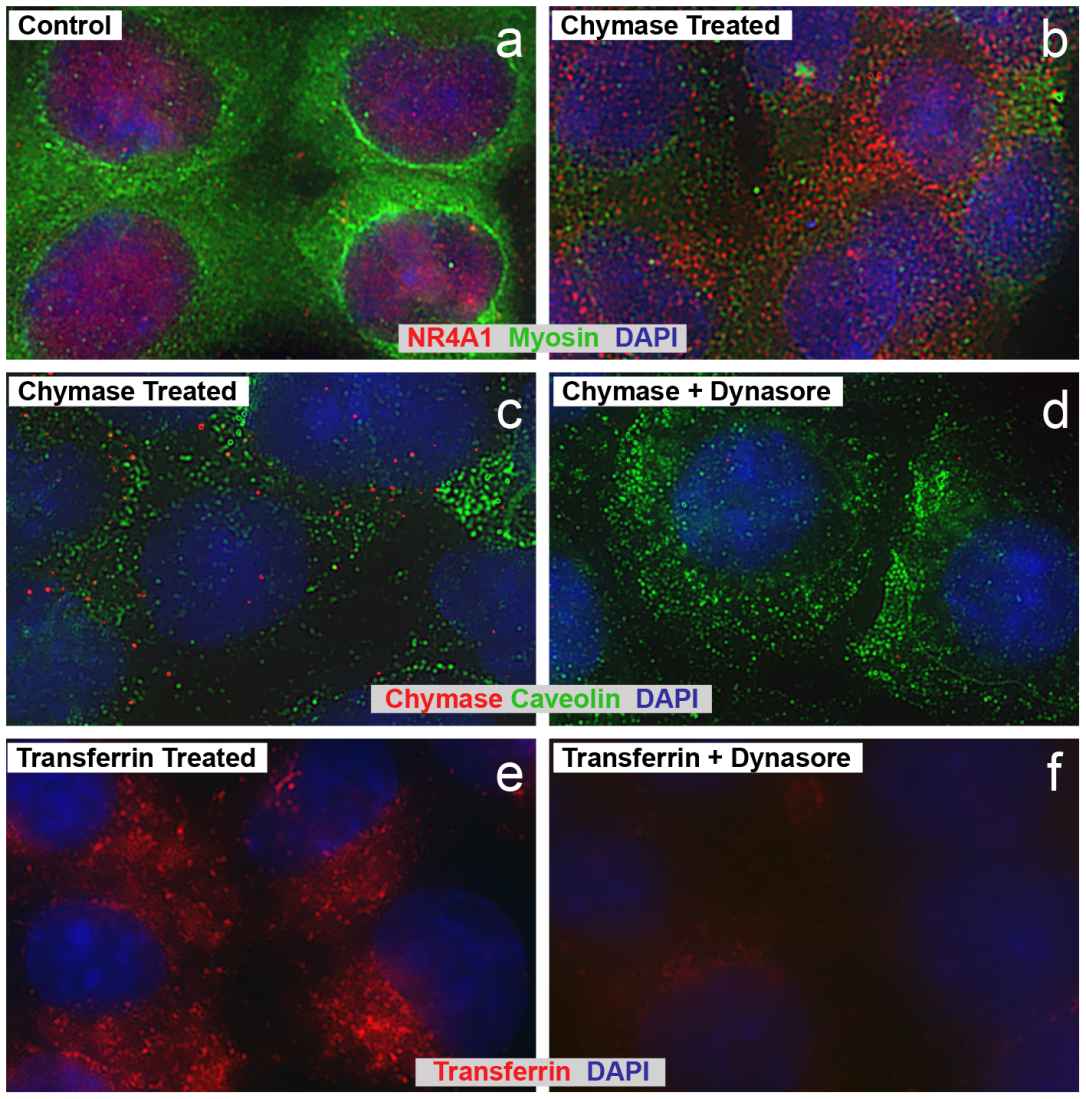
Chymase promotes cytoplasmic translocation of NR4A1 in HL-1 cells. Immunocytochemistry demonstrates NR4A1 nuclear location at control (non-chymase treated) in HL-1 cells (panel a). Chymase (2.5 µg/ml) treatment for 2 h induces NR4A1 (red) cytoplasmic translocation in HL-1 cells as well as myosin (green) disruption (panel b). Active chymase enters HL-1 cells and is prevented by Dynasore (panel c and d). There is a small amount of chymase at control in HL-1 cells (not shown) and marked entry into HL-1 cell nuclei and cytoplasm after treatment with chymase (5 µg/ml) for 2 h that is prevented by pre-treatment with Dynasore. Lack of co-staining with caveolin 3 (green) demonstrates that chymase is not transported *via* caveolae. Dynasore prevents transferrin uptake in HL-1 cells. There is marked entry of transferrin into HL-1 cell nuclei and cytoplasm after treatment with transferrin (5 µg/ml, panel e) for 2 h that is prevented by pre-treatment with Dynasore (panel f).

To more directly monitor the effects of chymase on NR4A1 expression and cytoplasmic translocation, HL-1 cells were treated with 2.5 µg/ml of chymase for 2 h and then examined by immunofluorescence microscopy. The results indicate that NR4A1 is localized to the nucleus and perinuclear areas in control cells (Figure 8a), but translocates to the cytoplasm after chymase treatment (Figure 8b). Furthermore, the myosin staining is dramatically reduced with chymase treatment (green staining in Figure 8b).

To monitor if chymase was taken up by cardiomyocytes, HL-1 cells were incubated with 2.5 µg/ml of chymase for 1 h and then processed for immunofluorescence microscopy. The results shown in Figure 8c indicate that chymase is taken up by the cells very efficiently. To test if the chymase was taken up through an endocytic process that involved dynamin, a GTPase for clathrin-dependent and caveolin-dependent endocytosis [25], a dynamin inhibitor, dynasore [26] was used to block this process. The results indicate that the dynasore treatment dramatically blocked chymase uptake (Figure 8d). Furthermore, chymase did not co-localize with caveolin (Figure 8c), suggesting that uptake was not through this pathway. To confirm that the dynasore was working, transferrin uptake through clathrin-coated pits was examined in untreated (Figure 8e) and dynasore-treated cells (Figure 8f), demonstrating that the dynasore effectively blocked transferrin uptake through this pathway in HL-1 cells.

**Figure 9:**
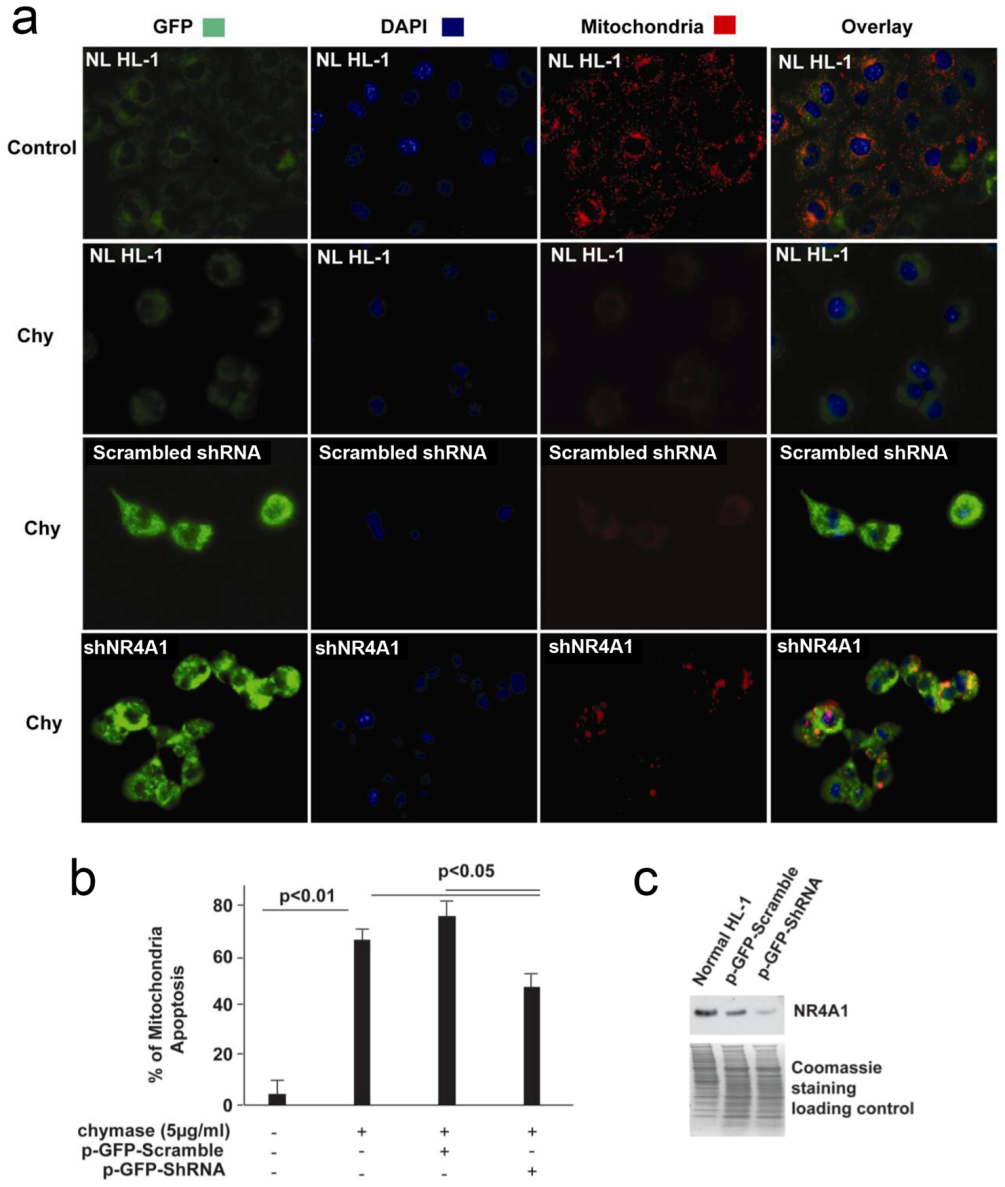
Knockdown of NR4A1 significantly attenuates chymase-induced mitochondria (Mt) apoptosis. (a) Immunofluorescence pattern of HL-1 cells transfected with p-GFP-shNR4A1 and scrambled p-GFP-shRNA for 5 days, loaded with dye (MitoCapture) and then treated with 5 µg/ml chymase (chy) for 2 h. Chymase treatment induces Mt apoptosis that is detected by loss of an electrochemical gradient and the inability to take up the MitoCapture dye. Normal Mt; bright red; Apoptotic Mt: little or no red staining. Bright green is the green fluorescent protein (GFP) and indicates that the cells were transfected with either p-GFP-shNR4A1 or scrambled p-GFP-shRNA. Cells without transfection and chymase treatment are used as the control. (b) Percentage of Mt apoptosis in chymase treated HL-1 cells, HL-1 cells transfected with p-GFP-shNR4A1 and scrambled p-GFP-shRNA. (c) NR4A1 protein expression in normal HL-1 cells, HL-1 cells transfected with p-GFP-shNR4A1 and scrambled p-GFP-shRNA. NL: HL-1 cells without transfection.

To directly connect chymase with NR4A1-induced mitochondrial degradation, HL-1 cells were transfected with p-GFP-shNR4A1 and scrambled p-GFP-shRNA for 5 days and then all three groups (Figure 9a, row 2–4) were treated with 5 µg/ml chymase for 2 hr. Figure 9 shows 4 immunohistochemical columns staining GFP, DAPI, mitochondria, and overlay of GFP/DAPI/mitochondria. The results depicted in Figure 9a indicate that the mitochondrial dye uptake in column 3 is compromised in the chymase treated cells compared to the normal controls, indicating that the mitochondrial electrochemical gradient had dissipated in the chymase treated cells. Furthermore, knockdown of NR4A1 with shRNA reversed the chymase effect on mitochondria in column 3 of chymase treated cells, whereas a scrambled shRNA control did not, suggesting that chymase was inducing this effect through the actions of NR4A1. To quantitate this, the number of apoptotic cells was counted under each of the conditions of control, chymase, chymase with scrambled shRNA, and chymase with shNR4A1. These results indicate that the knockdown in Figure 9b partially blocked the apoptosis effect. The amount of NR4A1 knockdown is shown in Figure 9c with a western blot that demonstrates the decrease in NR4A1 in shRNA treated cells.

## Discussion

With acute coronary artery occlusion, reperfusion triggers mast cell degranulation, releasing mast cell chymase and other mediators into the interstitium. Chymase inhibition has been shown to attenuate myocardial infarction after acute ischemia reperfusion. However, the mechanism is not fully understood. Here, we report a novel finding of chymase entry into the cardiomyocyte and the expression and translocation of an orphan nuclear receptor NR4A1 that is prevented by pretreatment with a chymase inhibitor (CI).

In a clinically relevant dog model, there is low ISF chymase activity at baseline that increases 3–4-fold after 60 min occlusion and 100 min reperfusion in the I/R region, and CI pretreatment reduces this significantly. Unexpectedly, ISF chymase activity is also increased in the non-I/R zone as measured by conversion of the chymase specific substrate for Ang I using *in vivo* microdialysis of the heart. This may be due the dynamic nature of the ISF space that allows for the diffusion of mast cell-derived chymase from the ischemic to the non-ischemic regions. In addition, a global increase of sympathetic drive to the heart in response to ischemia stress is known to activate mast cell degranulation due to the release of neurotransmitters in non-ischemic myocardium [27].

Despite the significant increase of ISF chymase activity in both ischemic and non-ischemic regions, the severe cardiomyocyte injury occurs only in the I/R area. *In vivo*, chymase is activated by dipeptidyl peptidase and is susceptible to inhibition by serine protease inhibitors, which may differ in the I/R and non-I/R areas [28]. Additionally, the synergy with oxidative and inflammatory extracellular milieu of the I/R region may explain the destructive effects of chymase in the I/R region. Indeed, MMP-9 activity, which is not increased in the non-I/R region, is markedly increased in the I/R region and significantly decreased by CI (Figure S1). Thus, MMP-9 from infiltrated polymorphonuclear and other inflammatory cells along with the oxidative I/R milieu may also help explain why there is severe damage in I/R but not non-I/R region.

Chymase has been previously shown to cause apoptosis through degradation of fibronectin and loss of FAK and Akt signaling [10]. In the current study, CI prevents laminin breakdown and FAK dephosphorylation in the I/R region, and significantly reduces cTnl release. *In vitro* studies of adult dog cardiomyocytes plated on laminin demonstrate that after just 2 h of chymase, there is extensive perinuclear lysosomal autophagic vacuoles and mitochondrial degeneration that is difficult to attribute to chymase acting solely on the cell surface. To further investigate the mechanism of chymase-mediated on I/R injury, we performed a gene microarray on the I/R myocardium from vehicle and CI treated dog hearts using the non-I/R ischemic region of the same heart and the normal heart as the double controls. This uncovered the new finding of a chymase effect on the expression and translocation of an orphan nuclear receptor NR4A1, which is known to be associated with cell death [14]–[16].

NR4A1 was originally identified as an immediate early response gene to a variety of extracellular stimuli. Recently it has been recognized as an important regulator for cell death by translocation from the nucleus to cytoplasm [15]. The first identification of NR4A1 (Nur77) in the regulation of cardiomyocyte death comes from a mouse model subjected to 50 min of cardiac ischemia followed by 50 min and 120 min reperfusion [16]. In this study, NR4A1 is located in the nuclei of cardiomyocytes under normal conditions. I/R induces mitochondrial translocation of NR4A1 (Nur77) that is essential for pro-apoptotic effects. In a similar fashion in the dog heart, I/R induces a marked increase of NR4A1 mRNA and protein expression and cytoplasmic translocation in those cardiomyocytes with severe mitochondrial degradation especially in the perinuclear region as demonstrated in TEMs. We further demonstrate that inhibiting ISF chymase significantly attenuates NR4A1 expression and its cytoplasmic translocation as well as I/R injury.

The transcriptional regulation of NR4A1 with CI suggests a chymase and NR4A1 connection that is supported by the results demonstrating that *in vitro* knockdown of NR4A1 in the HL-1 cells significantly reduces chymase-induced mitochondrial degradation. Although numerous studies have investigated the mechanisms of NR4A1 activation, signals that connect the extracellular stimuli with the activation of this orphan nuclear receptor are largely unknown. Recently, activation of NR4A1 has been identified as the converging point of signal transductions of the neurokinin 1 receptor (NK1R1) and insulin-like growth factor 1 (IGF1R) receptor. Both of these induce non-apoptotic cell death with autophagy features [29]. It has also been shown that the translocation of NR4A1 from the nucleus to the cytoplasm requires both activated JNK and MEKK1 and is inhibited by Akt activation [30]. In addition to NR4A1, gene microarray also demonstrates that I/R induces a marked increase of ATF3, which is reduced more than 50% by CI pretreatment. ATF3 has been shown to be an ER-stress marker in response to a variety of stress conditions in many different tissues including the heart [31]–[34]. Indeed, TEM demonstrates that I/R induces morphological changes in rough ER (rER) together with the loss of mitochondria, particularly in the perinuclear area of cardiomyocytes, which is attenuated by CI [33], [34]. The suppression of ATF3 expression by CI further supports the idea that chymase plays an active role in cardiomyocyte stress during acute I/R. These findings raise the intriguing questions: how does extracellular chymase initiate these intracellular events, and what is the connection between ISF chymase and NR4A1 activation in the cardiomyocytes? Of great interest is the new finding of a large amount of intracellular chymase in cardiomyocytes, in particular the nucleus, of the I/R region as monitored by immunohistochemistry.

To connect chymase to NR4A1 and cell death *in vivo*, the current study demonstrates that chymase added to HL-1 cells causes NR4A1 translocation and mitochondrial degradation, which is prevented by knockdown of NR4A1. As in the *in vivo* results during I/R, chymase gains access and increases in the HL-1 cell cytoplasm and nucleus. Chymase does not co-stain with caveolae, which are known to shuttle cargo from outside the cell. We also show that chymase enters HL-1 cells in a dynamin-dependent fashion, consistent with classic receptor-mediated endocytosis. These experiments do not rule out a requirement of surface receptor versus a proteoglycan or fibronectin requirement for endosomal entry of chymase. However, at this point we speculate that the most likely mechanism is a fibronectin/laminin binding of chymase to the cell surface since they both are substrates for chymase. Taken together, these results suggest a proteoglycan/fibronectin endocytic uptake mechanism similar to that proposed for neutrophil elastastase [35], [36], granzyme B [37], and myeloperoxidase [38].

The chymase inhibitor used in these studies was related to TEI-E00548 that has previously been described [39]. TEI-E00548 was shown to inhibit hamster chymase *in vitro* (k_i_ = 30.6 nM), with little effect on other serine proteases including trypsin, chymotrypsin, elastase, and cathepsin G, and moreover, did not inhibit ACE [12]. The inhibitor used in this study, TEI-F00806, is specific for chymase as well, but is approximately 3 times more potent than TEI-E00548 [12]. One limitation of the present study, however, was that there was no assessment of this CI on a more prolonged reperfusion on myocardial infarction size and LV function. Nevertheless, a similar short period of I/R in the pig demonstrates a significant decrease in infarct size using an intravenous dose of CI initiated at the time of reperfusion [13]. Future studies in dogs in a clinically relevant protocol of intravenous CI with prolonged reperfusion must determine whether the drug effects translate into improvement in LV function, arrhythmias and survival.

The results of the current investigation provide novel data that explain the multidimensional efficacy of chymase inhibitors in I/R that can now be attributed to chymase entry into the cardiomyocyte. Chymase mRNA is not increased after the short period of I/R, further supporting the contention that the mechanism of injury is largely due to activation of chymase released from resident and infiltrating mast cells. The effect of CI on apoptosis-related genes, coupled with the increase in nuclear chymase and NR4A1 translocation, suggests direct chymase-mediated effect on transcription and/or indirect effect through chymase-mediated intracellular Ang II formation. The latter mechanism in particular is especially important because angiotensin-converting enzyme inhibition or Ang II receptor blockade would not have a direct effect on intracellular Ang II formation because their Ang II effects are exerted on the cell surface.

## Supporting Information

Figure S1 **CI decreases MMP-9 activity in LV I/R region and release of MMP-9 protein.** (a, b) Gel zymography demonstrates a significant increase in LV MMP-9 activity in I/R region (n = 6) vs. normal (NL, n = 6) LV and LV non-I/R (n = 6), which is restored to normal levels by CI.
(TIF)

Table S1 **Hemodynamic changes during ischemia reperfusion.**
(DOCX)
