# Supplementary figures and images for: Chymase Mediates Injury and Mitochondrial Damage in Cardiomyocytes during Acute Ischemia/Reperfusion in the Dog
Source: PLoS One. 2014 Apr 14;9(4):e94732. doi: 10.1371/journal.pone.0094732 (PMC3986229; doi:10.1371/journal.pone.0094732)

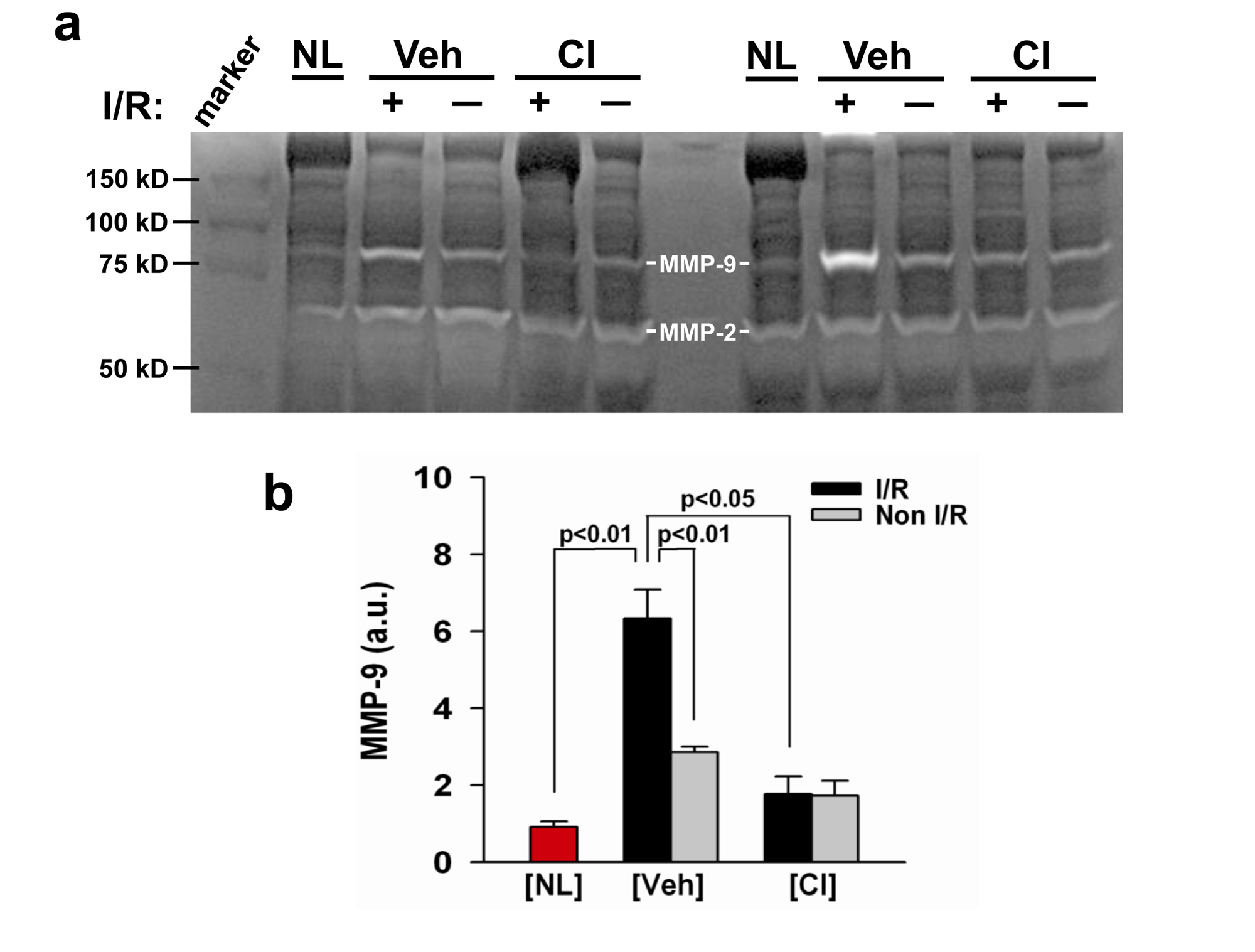

Supplement: Figure S1 — CI decreases MMP-9 activity in LV I/R region and release of MMP-9 protein. (a, b) Gel zymography demonstrates a significant increase in LV MMP-9 activity in I/R region (n = 6) vs. normal (NL, n = 6) LV and LV non-I/R (n = 6), which is restored to normal levels by CI. (TIF) [file pone.0094732.s001.tif]
