# Supplementary material for: Chymase Mediates Injury and Mitochondrial Damage in Cardiomyocytes during Acute Ischemia/Reperfusion in the Dog
Source: PLoS One. 2014 Apr 14;9(4):e94732. doi: 10.1371/journal.pone.0094732 (PMC3986229; doi:10.1371/journal.pone.0094732)
Supplement: Table S1 — Hemodynamic changes during ischemia reperfusion. (DOCX) [file pone.0094732.s002.docx]

| **Table S1. Hemodynamic changes during Ischemia Reperfusion** | | | | | | |
| --- | --- | --- | --- | --- | --- | --- |
|  | **Systolic Blood Pressure** | | **Diastolic Blood Pressure** | | **Heart Rate** | |
| **N=12** | **Vehicle** | **Chymase Inhibitor** | **Vehicle** | **Chymase Inhibitor** | **Vehicle** | **Chymase Inhibitor** |
| **Start I/R** | 106±21 | 100±25 | 66±15 | 62±18 | 110±8 | 105±8 |
| **End I/R** | 101±20 | 83±21* | 58±13 | 46±14* | 114±14 | 114±19 |
